# Development of a Prognostic Scoring System for Tracheostomized Patients Requiring Prolonged Ventilator Care: A Ten-Year Experience in a University-Affiliated Tertiary Hospital

**DOI:** 10.3390/medicina60020280

**Published:** 2024-02-06

**Authors:** Hyojin Jang, Wanho Yoo, Hayoung Seong, Saerom Kim, Soo Han Kim, Eun-Jung Jo, Jung Seop Eom, Kwangha Lee

**Affiliations:** 1 Division of Pulmonary, Allergy, and Critical Care Medicine, Department of Internal Medicine, Pusan National University Hospital, Busan 49241, Republic of Korea; hyoding2@pusan.ac.kr (H.J.); lcc2202@pusan.ac.kr (W.Y.); goldskygod7@pnuh.co.kr (H.S.); kimsaerom03@pusan.ac.kr (S.K.) kshyjt1004@gmail.com (S.H.K.); ej-jo@pusan.ac.kr (E.-J.J.); ejspulm@gmail.com (J.S.E.); 2 Biomedical Research Institute, Pusan National University Hospital, Busan 49241, Republic of Korea; 3 Department of Internal Medicine, School of Medicine, Pusan National University, Busan 49241, Republic of Korea

**Keywords:** mechanical ventilation, prognosis, mortality, intensive care unit

## Abstract

*Background and Objectives*: This study aimed to assess the value of a novel prognostic model, based on clinical variables, comorbidities, and demographic characteristics, to predict long-term prognosis in patients who received mechanical ventilation (MV) for over 14 days and who underwent a tracheostomy during the first 14 days of MV. *Materials and Methods*: Data were obtained from 278 patients (66.2% male; median age: 71 years) who underwent a tracheostomy within the first 14 days of MV from February 2011 to February 2021. Factors predicting 1-year mortality after the initiation of MV were identified by binary logistic regression analysis. The resulting prognostic model, known as the tracheostomy-ProVent score, was computed by assigning points to variables based on their respective ß-coefficients. *Results*: The overall 1-year mortality rate was 64.7%. Six factors were identified as prognostic indicators: platelet count < 150 × 10^3^/μL, PaO_2_/FiO_2_ < 200 mmHg, body mass index (BMI) < 23.0 kg/m^2^, albumin concentration < 2.8 g/dL on day 14 of MV, chronic cardiovascular diseases, and immunocompromised status at admission. The tracheostomy-ProVent score exhibited acceptable discrimination, with an area under the receiver operating characteristic curve (AUC) of 0.786 (95% confidence interval: 0.733–0.833, *p* < 0.001) and acceptable calibration (Hosmer–Lemeshow chi-square: 2.753, df: 8, *p* = 0.949). Based on the maximum Youden index, the cut-off value for predicting mortality was set at ≥2, with a sensitivity of 67.4% and a specificity of 76.3%. *Conclusions*: The tracheostomy-ProVent score is a good predictive tool for estimating 1-year mortality in tracheostomized patients undergoing MV for >14 days. This comprehensive model integrates clinical variables and comorbidities, enhancing the precision of long-term prognosis in these patients.

## 1. Introduction

In cases of critical illness, a subset of patients often necessitates extended mechanical ventilation (MV), presenting intricate clinical profiles [1,2]. Prolonged MV is associated with increased mortality rates, substantial physical and emotional morbidities, and considerable healthcare costs. Gaining insight into the factors influencing long-term outcomes in these patients is crucial for designing personalized treatment strategies, resource allocation, and determining comprehensive end-of-life care [3,4].

Prognostic models designed for patients requiring prolonged MV have been developed [5,6,7]. One notable example is the Prognosis for Prolonged Ventilation (ProVent) scoring system, crafted to estimate 1-year mortality in patients undergoing MV for a minimum of 21 days [5,6]. The validity and applicability of this model have been substantiated [8,9,10,11]. This has led to the creation of the ProVent 14 model, derived from demographic and clinical characteristics collected on day 14 for patients requiring more than 14 days of MV [7,12]. However, uncertainties persist regarding the adaptability of these models in diverse intensive care unit (ICU) contexts. Furthermore, due to the omission of various critical clinical variables and underlying comorbidities in these models, there is a recognized need for more comprehensive extended models [10,13].

Many patients receiving prolonged MV undergo a tracheostomy. This procedure offers several advantages, including enhanced patient comfort, reduced reliance on sedative agents, accelerated MV weaning, lower incidence of nosocomial pneumonia, and shorter hospital stays [14]. These benefits suggest that an early tracheostomy may have effects on clinical outcomes in patients receiving MV attention [15,16,17,18]. The prospect that patients with long-term MV and tracheostomy may exhibit distinct outcomes underscores the necessity for tailored prognostic models for this specific population.

The present study describes the construction of a scoring model based on clinical variables, underlying comorbidities, and demographic characteristics. The primary aim of this model was to predict 1-year mortality in patients receiving MV for >14 days and undergoing tracheostomy. This approach may enhance prognostic accuracy in this patient subgroup.

## 2. Materials and Methods

### 2.1. Study Design and Patient Selection

This retrospective observational study was conducted at a single center, focusing on individuals admitted to a 12-bed adult respiratory ICU in a university-affiliated tertiary care hospital with a nurse-to-bed ratio of 1:3, within a facility boasting 1100 beds. The ICU strictly adhered to therapeutic guidelines aligned with a lung-protective ventilator strategy [19].

The study enrolled adult patients (≥18 years) admitted to the respiratory ICU between 1 February 2011, and 31 January 2021. Inclusion criteria encompassed patients receiving mechanical ventilation (MV) for over 14 days post-endotracheal intubation and undergoing a tracheostomy within the initial 14 days of MV [7,12,13]. Tracheostomy decisions were made by physicians for patients anticipated to require prolonged mechanical ventilation. Excluded from the study were individuals with pre-existing chronic conditions requiring invasive mechanical ventilation prior to ICU admission, those under the age of 18, and patients with irreversible brain injury, neuromuscular disorders, or inadequate medical documentation. This stringent and thorough patient selection process aimed to enhance the study’s internal validity and ensure a more homogeneous study population, contributing to the robustness of the findings and their applicability to similar clinical settings.

### 2.2. Outcome and Data Collection

The primary focus of this study was the mortality rate one year after the 14th day of mechanical ventilation. Demographic and clinical information, including age, gender, body mass index (BMI), primary diagnoses necessitating mechanical ventilation, duration of ventilation, length of stay in the ICU and hospital, as well as in-ICU and in-hospital mortality, were gathered retrospectively from electronic medical records (EMRs), ensuring a rich and detailed dataset for robust analysis. To gain a holistic understanding of the patients’ health status, factors, such as illness severity (APACHE II scores) [20], organ failure (SOFA score) [21], and underlying comorbidities (Charlson’s weighted index) [22] were assessed based on data obtained on the day of ICU admission. Additionally, 1-year mortality post-ICU admission was evaluated through the National Health Insurance Service database. This additional layer of data not only extends the temporal scope of the study but also facilitates a comprehensive analysis of the long-term outcomes beyond the immediate ICU period.

### 2.3. ProVent 14 Scores and Additional Variables

ProVent 14 scores were based on clinical variables obtained on day 14 of MV, including blood platelet count, hemodialysis need, and the requirement of vasopressor. Based on studies identifying prognostic factors for patients with long-term ventilator care [10,13,23], other laboratory and clinical varables were obtained on day 14 of MV, including leukocyte count, PaO_2_/FiO_2_ ratio, serum albumin and bilirubin concentrations, and delirium occurrence. Hemodialysis need was defined as any renal replacement therapy on or within 48 h of day 14 of MV. Delirium diagnosis was confirmed through psychiatric consultation and medication prescription.

### 2.4. Statistical Analysis

The median (interquartile range [IQR]) was used to express continuous variables, and comparisons were made through Mann–Whitney U tests. Categorical variables were presented as numbers (percentages) and analyzed using either χ^2^ or Fisher’s exact tests. Independent predictors of 1-year mortality were identified through stepwise logistic regression, and the coefficients were reported as positive natural numbers [6,7,12,13,24]. The tracheostomy-ProVent score, derived from these coefficients, was developed. Kaplan–Meier analysis, log-rank tests, receiver operation characteristic (ROC) curve analysis, area under the curve (AUC) determination, and model calibration assessment were employed for prognostic model evaluation. The AUCs were assessed in comparison to APACHE II and SOFA scores using the DeLong test [25]. Optimal cut-off values were established by maximizing Youden’s index [26]. Sensitivity, specificity, positive likelihood ratio, negative likelihood ratio, positive predictive value, and negative predictive value were then computed. Statistical significance was set at *p* < 0.05. SPSS version 24.0 and MedCalc version 22.007 were utilized for all analyses.

## 3. Results

### 3.1. Patient Characteristics

**Figure 1:**
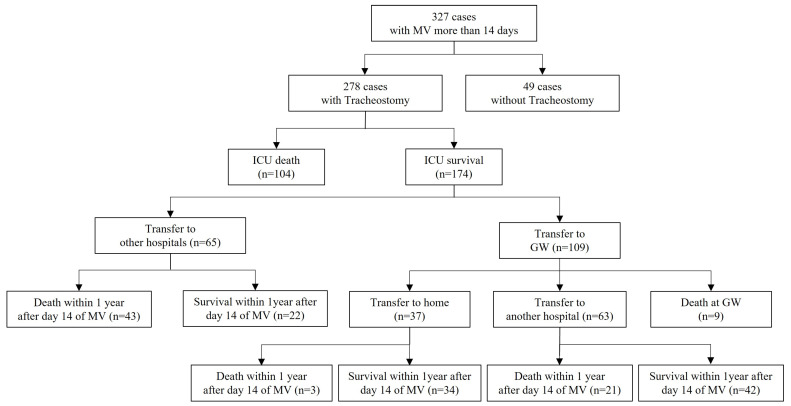
Study flow diagram. MV, mechanical ventilation; ICU, intensive care unit; GW, general ward.

Over the course of a decade, a total of 2024 patients underwent ventilator care, with 340 (16.7%) necessitating mechanical ventilation (MV) for over 14 days. Thirteen exclusions were made, with seven attributed to irreversible brain injury and six to incomplete medical records. The finalized study cohort comprised 327 patients who underwent MV for a duration surpassing 14 days (refer to Figure 1 for a visual representation).

In Table 1, a detailed overview of clinical characteristics and outcomes is provided for patients both with and without a tracheostomy during their hospitalization. Notably, patients without a tracheostomy displayed higher Sequential Organ Failure Assessment (SOFA) scores upon ICU admission, indicating a greater degree of organ dysfunction. Additionally, this group exhibited extended lengths of stay in both the ICU and hospital, underscoring the complexity and severity of their medical conditions. Furthermore, a compelling finding emerges as those without tracheostomy demonstrated a notably higher 1-year cumulative mortality rate compared to their counterparts who underwent a tracheostomy, shedding light on the potential impact of a tracheostomy on long-term survival outcomes.

### 3.2. Analysis of Tracheostomized Patients

Within the subset of tracheostomized patients, those aged 65 years or older who did not survive exhibited elevated prevalence rates of underlying comorbidities, specifically cardiovascular diseases, chronic lung diseases, hemato-oncologic diseases, chronic kidney diseases, and immunocompromised states. The non-survivors within this age group demonstrated heightened demands for vasopressors and hemodialysis, indicative of a more complex and severe clinical course. Moreover, notable differences in laboratory parameters were observed on day 14 of mechanical ventilation (MV), with non-survivors presenting lower platelet counts (<150 × 10^3^/μL) and PaO_2_/FiO_2_ ratios (≤200 mmHg) compared to their surviving counterparts. Additionally, albumin and bilirubin concentrations on day 14 of MV were lower in non-survivors than survivors (Table 2).

### 3.3. Prognostic Model for 1-Year Mortality in Tracheostomized Patients

**Figure 2:**
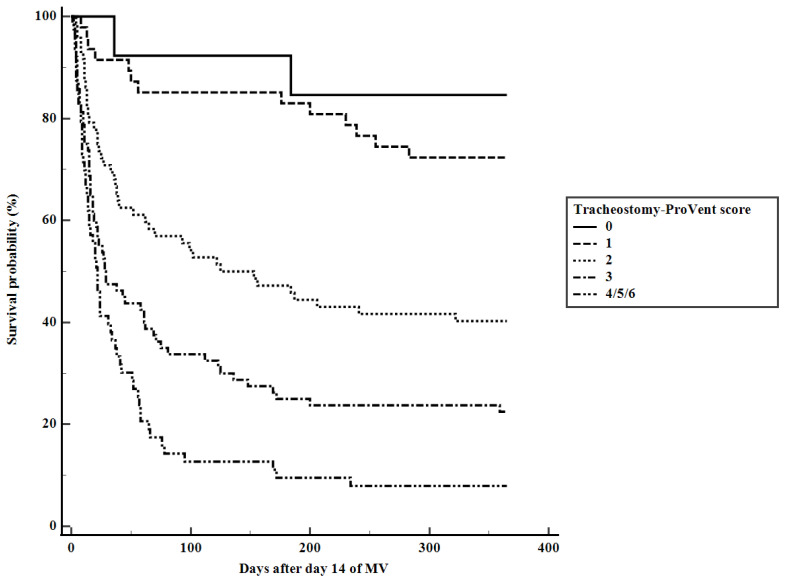
Kaplan–Meier survival curves of patients stratified according to tracheostomy-ProVent scores (Log-rank *p*-value < 0.001).

**Figure 3:**
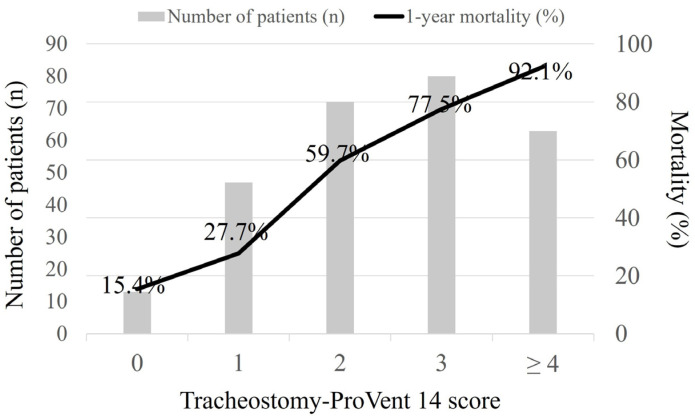
Numbers of patients sorted by tracheostomy-ProVent scores and their corresponding 1-year mortality rates.

**Figure 4:**
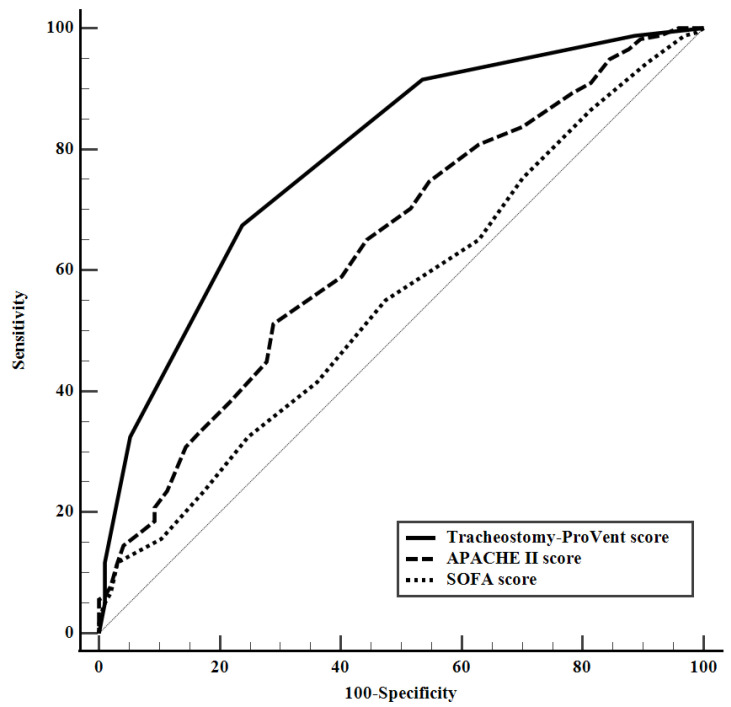
Comparison of receiver-operating characteristic curves (ROCs) for the ability of tracheostomy-ProVent scores (AUC 0.786, 95% CI 0.733–0.833), Acute Physiology and Chronic Health Evaluation (APACHE) II scores (AUC 0.644, 95% CI 0.5843–0.701), and Sequential Organ Failure Assessment (SOFA) scores (AUC 0.553, 95% CI 0.492–0.613) to predict 1-year mortality. Tracheostomy-ProVent scores demonstrated a significant superiority over both APACHE II (*p* = 0.0003) and SOFA scores (*p* < 0.0001).

Multivariate logistic regression identified six significant predictors of 1-year survival in tracheostomized patients. These included BMI ≤ 23.0 kg/m^2^, two underlying comorbidities (cardiovascular diseases and immunocompromised state), and three laboratory parameters on day 14 of MV (platelet count < 150 × 10^3^/μL, PaO_2_/FiO_2_ ≤ 200 mmHg, and albumin ≤ 2.8 g/dL) (Table 3). Logistic regression analysis revealed no significant variables influencing one-year survival when evaluating patients who did not undergo tracheostomy (Table 4). The tracheostomy-ProVent score, calculated from β-coefficients, demonstrated satisfactory discriminative ability (AUC = 0.786, 95% CI: 0.733–0.833) and excellent calibration (Hosmer–Lemeshow chi-square = 2.753, df = 8, *p* = 0.949). Figure 2 illustrates Kaplan–Meier survival curves, stratified based on the tracheostomy-ProVent score, while Figure 3 provides corresponding patient numbers and 1-year mortality rates. The designated cut-off value for mortality prediction (≥2) yielded a sensitivity of 67.4%, specificity of 76.3%, positive likelihood ratio of 2.8, negative likelihood ratio of 0.4, positive predictive value of 83.9%, and negative predictive value of 56.1%. Notably, the AUC of the tracheostomy-ProVent score demonstrated a significant superiority over both the APACHE II and SOFA scores, as illustrated in Figure 4.

## 4. Discussion

This study was designed to identify factors associated with 1-year mortality in tracheostomized patients requiring MV for >14 days. Many patients requiring extended MV have complicated clinical profiles, with outcomes reported to be better in tracheostomized than in non-tracheostomized patients, suggesting that a tracheostomy may improve patient prognosis. The strength of our study lies in its unique contribution to understanding the factors affecting long-term outcomes in this specific patient cohort.

Unlike prior inquiries [7,12,13], this study specifically targeted individuals admitted to a respiratory ICU. Notably, this patient cohort was characterized by advanced age and extended hospitalization, distinguishing them from participants in earlier investigations. Due to the unique attributes of this patient population, the evaluative capacity of the original ProVent 14 score in predicting 1-year mortality within this particular cohort remains unassessed. Distinct ICU environments and patient subsets may, therefore, affect the variability in long-term prognostic indicators.

Prognostic factors in the present patient cohort were identified through a composite approach, with variables including BMI, underlying comorbidities, and laboratory parameters on day 14 of MV. Although some of these indicators had been previously identified as prognostic factors [7,12,13], the present study identified additional distinctive prognostic factors. These findings suggest that the factors predictive of 1-year mortality might differ among populations of tracheostomized patients requiring MV for >14 days.

Pre-existing comorbidities have been found to affect the course of critical illnesses and eventual outcomes. The present analysis of tracheostomized patients requiring MV for >14 days showed that the rates of comorbidities, including cardiovascular, chronic lung, hemato-oncologic, and chronic kidney diseases, as well as immunocompromised states, were higher in non-survivors than in survivors. Likewise, the presence of pre-existing comorbidities was identified as a significant factor influencing the prognosis of critically ill patients [13]. Therefore, the occurrence of comorbidities should be recognized as a crucial long-term prognostic indicator for critically ill patients necessitating extended mechanical ventilation.

Other, as yet unidentified, factors may serve as valuable prognostic indicators in tracheostomized patients requiring MV for >14 days. Clinical variables found to be prognostically relevant in critically ill patients include chest radiographic findings, pH < 7.35, lactic acid concentrations > 2.0 mmol/L, and serum sodium concentrations ≥ 145 mmol/L [27,28,29]. In our study, pH exhibited variability on the 14th day of examination, while lactic acid could not be used as a variable since it was often not measured on the 14th day. Further research is necessary to pinpoint additional clinical variables that might serve as prognostic indicators for long-term outcomes in patients with a tracheostomy undergoing extended mechanical ventilation.

The tracheostomy-ProVent score described in this study and based on six prognostic factors was developed to enhance the ability to predict 1-year mortality in tracheostomized patients requiring extended MV. The ability of this score to predict outcomes was underscored by its satisfactory AUC. The AUC of this prognostic model was significantly higher than the AUCs of the APACHE II and SOFA scores. The tracheostomy-ProVent score’s threshold value, derived from the maximum Youden index, allows clinicians to categorize patients into different risk groups. The findings of this study hold the potential to influence clinical decision-making. Identifying factors linked to 1-year mortality in tracheostomized patients undergoing prolonged mechanical ventilation can pinpoint those at high risk. This understanding may contribute to refining treatment approaches, enhancing resource allocation, and fostering more inclusive discussions about end-of-life care with patients and their families.

Clinical outcomes in patients requiring MV can be influenced by factors other than clinical variables. These factors include the assessment of frailty, the perspectives of the patient’s family, communications between physicians and families, and the allocation of medical resources [30,31,32]. Furthermore, the presence and sophistication of critical care resources in university hospitals can significantly affect prognoses. Large-scale multicenter investigations that include socioeconomic and ethical factors, as well as clinical variables, are needed in the development of comprehensive prognostic models and may provide a more holistic understanding of patient outcomes.

Although the subset of patients without a tracheostomy who required MV for >14 days was smaller than that of patients who underwent a tracheostomy, the prognostic feasibility of both the original ProVent 14 score and the tracheostomy-ProVent score could not be evaluated in this group. Additionally, logistic regression analysis could not identify prognostic factors associated with 1-year mortality in the non-tracheostomized group, perhaps because of the limited number of patients available for analysis. Prognostic models tailored to non-tracheostomized patients requiring prolonged MV are, therefore, needed.

Several limitations were associated with this study. Firstly, we observed that a lower BMI was linked to 1-year mortality; nevertheless, the determined cutoff value fell within the healthy weight range. Consequently, further investigation with a substantial patient cohort is warranted to establish a more suitable cutoff value. Secondly, we identified both BMI and albumin as prognostic markers, serving as proxies for well-nourished patients. Nevertheless, these indicators could be more accurately reflected by daily caloric intake. Unfortunately, due to the retrospective observational design, we were unable to assess the daily caloric intake of all enrolled patients. Thirdly, the inclusion of patients from a single center could restrict the generalizability of the findings. Furthermore, the complex interplay of variables in critically ill patients, coupled with the potential impact of unmeasured factors, necessitates careful consideration when interpreting the study’s conclusions.

## 5. Conclusions

The present study emphasizes the ability of specific factors to predict 1-year mortality in tracheostomized patients requiring prolonged MV. The tracheostomy-ProVent score described in this study may be used to assess risk in this subset of critically ill patients. Multicenter studies and external validation are required to confirm these findings.

## Figures and Tables

**Table 1:** Baseline demographic and clinical characteristics of patients who did and did not undergo a tracheostomy.

Variable	Tracheostomy (*n* = 278)	Non-Tracheostomy (*n* = 49)	*p*-Value
Age, years	71 (61–77)	64 (54–76)	0.711
Male, n (%)	184 (66.2)	33 (67.3)	0.874
Body mass index (kg/m^2^) ^1^	22.0 (19.8–24.0)	22.0 (19.6–25.9)	0.480
Major diagnoses leading to MV, n (%)			
Pulmonary including pneumonia	204 (73.4)	34 (69.4)	0.563
Neurologic diseases	26 (9.4)	3 (6.1)	0.463
Infectious diseases other than pneumonia	22 (7.9)	8 (16.3)	0.060
Postoperative state	7 (2.5)	2 (4.1)	0.537
Trauma	5 (1.8)	0 (0.0)	0.344
Cardiovascular diseases	4 (1.4)	0 (0.0)	0.398
Others, n (%) ^2^	10 (3.6)	2 (4.1)	0.868
Severity of illness			
APACHE II score	20 (15.0–25.0)	20 (15–26)	0.366
SOFA score	7 (4.0–9.0)	9 (5–11)	0.041
Charlson’s comorbidity index	4 (3–6)	5 (3–6)	0.046
MV length of stay, days	25 (19–35)	17 (15–22)	0.238
Hospital length of stay, days	41.0 (27.0–69.3)	27.0 (17.5–46.0)	0.291
ICU length of stay, days	27.0 (20.0–39.3)	20.0 (16.0–24.0)	0.523
Mortality, n (%)			
ICU mortality	104 (37.4)	31 (63.3)	0.001
In-hospital mortality	113 (40.6)	32 (65.3)	0.001
1-year cumulative mortality	180 (64.7)	41 (83.7)	0.009

^1^ Body mass index was obtained for 275 tracheostomized and 47 non-tracheostomized patients. ^2^ Others included rhabdomyolysis (1), diabetic ketoacidosis (1), unknown drug intoxication (1), acute pancreatitis (1), and post-CPCR state due to variant angina (2), asphyxia (1), hemoptysis (2), and unknown cause (3). The numbers indicated in parentheses represent the number of patients with the respective medical condition. MV, mechanical ventilation; APACHE, Acute Physiology and Chronic Health Evaluation; SOFA, Sequential Organ Failure Assessment; ICU, intensive care unit;

**Table 2:** Baseline demographic and clinical characteristics of tracheostomized survivors and non-survivors.

Variable	Survivors (*n* = 98)	Non-Survivors (*n* = 180)	*p*-Value
Age ≥ 65 years	56 (57.1)	130 (72.2)	0.011
Clinical variables on day 14 of MV			
Requirement for vasopressors, n (%)	23 (23.5)	72 (40.0)	0.005
Requirement for hemodialysis, n (%)	5 (5.1)	25 (13.9)	0.024
Occurrence of delirium, n (%)	11 (11.2)	12 (6.7)	0.188
Laboratory data on day 14 of MV			
Platelet count < 150 × 10^9^ /L, n (%)	22 (22.4)	96 (53.3)	<0.001
Leukocytosis, n (%)	33 (33.7)	80 (44.4)	0.081
PaO_2_/FiO_2_ < 200 mmHg, n (%)	17 (17.3)	67 (37.2)	0.001
Albumin	2.7 (2.4–3.0)	2.5 (2.3–2.9)	0.002
Bilirubin	0.7 (0.4–1.1)	0.9 (0.5–1.7)	0.004
Underlying comorbidities, n (%)			
Diabetes mellitus	32 (32.7)	59 (32.8)	0.983
Cardiovascular diseases	14 (14.3)	62 (34.4)	<0.001
Chronic lung diseases	16 (16.3)	49 (27.2)	0.040
Neurologic diseases	28 (28.6)	36 (20)	0.105
Hemato-oncologic diseases	11 (11.2)	49 (27.2)	0.002
Chronic kidney diseases	5 (5.1)	27 (15)	0.013
Immunocompromised	2 (2.0)	25 (13.9)	0.001
Chronic liver diseases	11 (11.2)	15 (8.3)	0.429
Rheumatologic diseases	2 (2)	7 (3.9)	0.406

MV, mechanical ventilation; PaO_2_, partial pressure of oxygen in arterial blood; FiO_2_, fraction of inspiratory oxygen concentration.

**Table 3:** Logistic regression analysis of factors associated with 1-year mortality in patients who underwent a tracheostomy.

Variable	Unadjusted OR (95% CI)	*p*-Value	Adjusted OR (95% CI)	*p*-Value	β Value
Age ≥ 65 years	1.950 (1.164–3.267)	0.011			
BMI ≤ 23.0 kg/m^2^	2.159 (1.295–3.597)	0.003	2.145 (1.184–3.887)	0.012	0.763
Clinical variables on day 14 of ventilator care					
Requirement for vasopressors	2.174 (1.249–3.784)	0.006			
Requirement for hemodialysis	3.000 (1.110–8.106)	0.030			
Laboratory data on day 14 of ventilator care					
Platelet count < 150 × 10^3^/μL	3.948 (2.261–6.895)	<0.001	3.256 (1.736–6.106)	<0.001	1.180
Leukocytosis	1.576 (0.945–2.629)	0.082			
PaO_2_/FiO_2_ ≤ 200 mmHg	2.825 (1.544–5.168)	0.001	2.953 (1.488–5.862)	0.002	1.083
Albumin ≤ 2.8 g/dL	2.277 (1.353–3.834)	0.002	1.888 (1.016–3.510)	0.044	0.636
Underlying comorbidities					
Cardiovascular diseases	3.153 (1.656–6.002)	<0.001	2.945 (1.396–6.212)	0.005	1.080
Chronic lung diseases	1.917 (1.023–3.593)	0.042			
Hemato-oncologic diseases	2.958 (1.458–6.005)	0.003			
Chronic kidney diseases	3.282 (1.222–8.820)	0.018			
Immunocompromised	7.742 (1.793–33.423)	0.006	8.934 (1.898–42.055)	0.006	2.190

All six variables were included in the multivariate analysis using stepwise backward selection procedures (Hosmer–Lemeshow chi-square = 2.753, df = 8, p = 0.949). OR, odds ratio; CI, confidence interval; BMI, body mass index; PaO_2_, partial pressure of oxygen in arterial blood; FiO_2_, fraction of inspiratory oxygen concentration.

**Table 4:** Logistic regression analysis of factors associated with one-year mortality in patients without a tracheostomy.

Variable	Unadjusted OR (95% CI)	*p*-Value
Age ≥ 65 years	1.750 (0.369–8.302)	0.481
Clinical variables on day 14 of MV		
Requirement for vasopressors	2.604 (0.546–12.428)	0.230
Requirement for hemodialysis	0.618 (0.103–3.719)	0.599
Laboratory data on day 14 of MV		
Platelet count < 150 × 10^9^ /L	5.200 (0.929–29.094)	0.061
Leukocytosis	0.640 (0.140–2.930)	0.565
Underlying comorbidities, n (%)		
Cardiovascular diseases	2.897 (0.321–26.158)	0.344
Chronic kidney diseases	1.321 (0.128–13.656)	0.815

OR, odds ratio; CI, confidence interval; MV, mechanical ventilation.

## Data Availability

The data supporting the conclusions of this study can be obtained by contacting the corresponding author upon request. However, these data are not publicly accessible due to privacy or ethical constraints.
